# Dynamic Cultivation of Mesenchymal Stem Cell Aggregates

**DOI:** 10.3390/bioengineering5020048

**Published:** 2018-06-19

**Authors:** Dominik Egger, Carla Tripisciano, Viktoria Weber, Massimo Dominici, Cornelia Kasper

**Affiliations:** 1 Department of Biotechnology, University of Natural Resources and Life Sciences, Muthgasse 18, 1190 Vienna, Austria; dominik.egger@boku.ac.at; 2 Christian Doppler Laboratory for Innovative Therapy Approaches in Sepsis, Danube University Krems, Dr.-Karl-Dorrek-Straße 30, 3500 Krems, Austria; carla.tripisciano@donau-uni.ac.at (C.T.); viktoria.weber@donau-uni.ac.at (V.W.); 3 Division of Oncology, Department of Medical and Surgical Sciences for Children & Adults, University-Hospital of Modena and Reggio Emilia, Via Università 4, 41121 Modena, Italy; massimo.dominici@unimore.it; 4 Technopole of Mirandola TPM, 41037 Mirandola, Modena, Italy

**Keywords:** mesenchymal stem cells, aggregates, spheroids, dynamic cultivation, bioreactor cultivation, extracellular vesicles, therapeutic potential, scaffold-free

## Abstract

Mesenchymal stem cells (MSCs) are considered as primary candidates for cell-based therapies due to their multiple effects in regenerative medicine. Pre-conditioning of MSCs under physiological conditions—such as hypoxia, three-dimensional environments, and dynamic cultivation—prior to transplantation proved to optimize their therapeutic efficiency. When cultivated as three-dimensional aggregates or spheroids, MSCs display increased angiogenic, anti-inflammatory, and immunomodulatory effects as well as improved stemness and survival rates after transplantation, and cultivation under dynamic conditions can increase their viability, proliferation, and paracrine effects, alike. Only few studies reported to date, however, have utilized dynamic conditions for three-dimensional aggregate cultivation of MSCs. Still, the integration of dynamic bioreactor systems, such as spinner flasks or stirred tank reactors might pave the way for a robust, scalable bulk expansion of MSC aggregates or MSC-derived extracellular vesicles. This review summarizes recent insights into the therapeutic potential of MSC aggregate cultivation and focuses on dynamic generation and cultivation techniques of MSC aggregates.

## 1. Therapeutic Relevance of Mesenchymal Stem Cells

In the field of regenerative medicine, mesenchymal stem cells (MSCs) are considered primary candidates for cellular therapies and tissue engineering. They can be harvested from a variety of tissues, such as bone marrow, adipose tissue, or umbilical cords. Minimal criteria for the characterization of human MSCs defined in a position paper by the International Society for Cellular Therapies (ISCT) comprise plastic adherence, trilineage differentiation (adipogenic, chondrogenic, osteogenic), as well as a specific surface marker expression profile (CD105^+^, CD73^+^, CD90^+^, CD14^−^, CD19^−^, CD34^−^, CD45^−^ and HLADR^−^) [1]. Although MSCs might display similar properties across different species this review considers only results from research on human MSCs.

The regenerative potential of MSCs is not limited to their ability to differentiate into adipocytes, chondrocytes, and osteoblasts, as indicated by a number of studies reporting MSC differentiation into neurons [2], cardiomyocytes [3], and corneal epithelial cells [4] along with effects related to injury repair, such as migration to injury sites [5,6], angiogenesis [7] and anti-scarring effects [8]. MSCs display immunomodulatory and anti-inflammatory properties mediated by cellular cross talk [9] or by secretion of trophic factors, such as transforming growth factor-β (TGF-β), IL-6, prostaglandin E2 (PGE2), platelet-derived growth factor (PDGF), insulin-like growth factor (IGF), fibroblast growth factor (FGF), epidermal growth factor (EGF), stromal cell-derived factor 1 (SCDF-1), and vascular endothelial growth factor (VEGF) [10,11]. MSCs have been applied in a number of clinical trials with promising results for the treatment of graft-versus-host disease (GvHD), myocardial injuries, as well as bone and cartilage defects [12], and further investigations were conducted in the context of pulmonary disease, ischemic stroke, liver disease, and diabetes [13,14].

### Stem Cell-Derived Extracellular Vesicles

It is generally recognized that MSCs exert their therapeutic effects via the secretion of paracrine factors and stimulation of host cells rather than via direct engraftment and cell replacement, and there is increasing evidence for the significance of MSC-derived extracellular vesicles (EVs) in this context [15,16]. EVs are small phospholipid vesicles released from a wide variety of cell types, which are commonly classified into exosomes (30–100 nm; intraluminal vesicles originating from multivesicular bodies), microvesicles (100–1000 nm, released from the plasma membrane), and apoptotic bodies (1–5 µm) according to their biogenesis and size. Exosomes preferentially expose molecules related to endosomal trafficking, such as tetraspanins (CD9, CD63, and CD81) or Alix, while microvesicles are enriched with surface markers derived from their parent cells, such as CD73 and CD90 for MSC-derived EVs. However, as there are considerable overlaps in both, size and marker profiles of exosomes and microvesicles, and their precise separation is not yet technically feasible, the use of the collective term “extracellular vesicles” has been recommended by the International Society for Extracellular Vesicles (ISEV) [17].

EVs are central mediators in a number of physiological processes, including intercellular communication, cell signaling, and maintenance of tissue homeostasis, but also in pathological settings, such as inflammation and cancer. They can be internalized by a variety of cell types and transfer bioactive molecules (e.g., cytokines, growth factors, as well as coding and regulatory genomic material, such as mRNA, miRNA, siRNA, piRNA) to their recipient cells [18]. The structure of EVs protects their cargo from enzymatic degradation, and the presence of membrane proteins enables tailored delivery to their target cells [19]. Additionally, the solubility, local availability, and bioactivity of specific factors can be enhanced by their association with EV membranes [20].

The functional consequences of EV-mediated transfer of bioactive molecules include the induction, amplification, and regulation of immune responses, which has sparked considerable interest in the application of EVs as therapeutic agents [21]. In fact, MSC-derived EVs have been shown to recapitulate the ability of their parent cells to deliver signals related to immune regulation [22,23]. In vitro data indicate that (1) MSC-derived EVs can target a range of adaptive and innate immune cells [24], that (2) EVs from different MSC sources employ different immunomodulatory mechanisms [25] and can have different effects on their target cells [15,26], and that (3) MSC-derived EVs may mediate both, immunosuppressive properties and enhanced immune responses [27]. While these findings show that MSC-derived EVs can recapitulate the ability of their parent cells to deliver signals related to immune regulation [22,23], several studies have provided evidence that MSC-derived EVs do not fully reflect the effects exerted by their parent cells [28,29,30]. These diverging findings may at least partially result from different experimental approaches, such as different culture conditions of MSCs, isolation, and standardization of MSC-derived EV populations, as well as variable in vitro co-culture conditions, highlighting the requirement for standardized protocols [17]. The tissue source, the isolation, as well as the culture conditions can indeed influence the biological activity of MSC-derived EVs, as recently reviewed [14]. As an example, proteomic analysis of EVs from bone marrow-derived MSCs revealed significantly increased expression of proteins associated with angiogenic signaling under ischemic conditions [31] and hypoxic preconditioning enhanced the release of EVs enriched in miRNAs involved in wound healing [32].

In addition to the in vitro data, a number of studies have investigated the therapeutic effects of MSC-derived EVs in vivo using animal disease models of myocardial infarction, stroke, kidney failure, and liver fibrosis, as summarized in [33]. A recent review of controlled trials using MSC-derived EVs concluded that their administration to animals was safe and could contribute to improved organ function following injury [34]. Two clinical studies in humans using MSC-derived EVs have been reported so far. In the first case, a patient suffering from steroid refractory GvHD was successfully treated with MSC-derived EVs. Although it remains unclear which fraction or components of the EV preparation were responsible for the anti-inflammatory effects, the study suggests that MSC-derived EVs modulated the response of patients’ immune cells [35]. In the second study, patients suffering from chronic kidney disease and administered twice with cord-blood MSC-derived EVs showed improved kidney function and beneficial modulation of inflammatory markers, i.e., increase of TGF-β1 and IL-10 and decrease of TNF-α levels in response to treatment [36].

This suggests that MSC-derived EVs could represent an alternative to whole cell therapies. They may have a superior safety profile as compared to whole cells, and due to their size in the nanometer range, injected EVs can circulate through capillaries without entrapment by filter organs [37] and can cross biological barriers [38]. Their perceived capacity to survive and retain their activity during storage further supports MSC-derived EVs as a promising alternative tool for cell-free therapies [39,40,41]. As products of viable active stem cells, MSC-derived EVs are classified as “biological medicinal products”, but aspects such as information on the active substances and the mechanism of action are still not fully elucidated. Based on the criteria for “high risk medicinal products” (HRMPs) classification, such as lack of knowledge on the mechanisms of action, no clear understanding of the target, and limited relevance of animal models, EV-based therapeutics might be categorized as such, leading to the demand of strict pre-clinical safety tests. To this purpose, the ISEV has discussed aspects concerning safety and regulatory matters to be taken into account for clinical application or medicinal manufacturing, pointing out that, as allogenic EVs are routinely transfused within blood products and there is little evidence on adverse effects and as the response to previous treatments with autologous and allogenic MSCs has been positive, MSC-EVs should not be considered as HRMPs [42].

## 2. 3D Aggregate Cultivation of MSCs

While biological, chemical, physical, and mechanical cues can profoundly influence cellular characteristics, commonly used MSC cultivation conditions, such as 2D cultivation on plastic surfaces under static conditions are far from representing the physiological environment of these cells. To reflect physiological conditions in vitro, cells can either be cultivated on 3D matrices or in a scaffold-free manner as cellular aggregates, often referred to as spheroids. While the general term ‘aggregate’ describes any multicellular entity of condensed cells, the term ‘spheroid’ refers to spherical cellular aggregates. In embryonic stem cell research, where aggregate cultivation has been used since decades, it is also referred to as organoid culture [43].

**Figure 1:**
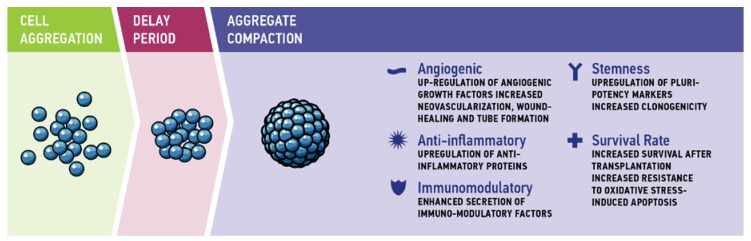
Three-step aggregation process: during the first phase of cell aggregation, cadherin–cadherin interactions and integrin binding to extracellular matrix proteins mediate the first cell–cell contacts. After a delay period of reorganization, aggregate compaction is mediated by cadherins.

The dynamics of spheroid formation comprises three stages. Cadherin-cadherin interaction and integrin binding to extracellular matrix (ECM) proteins mediate first cell-cell contacts to form loose cellular aggregates. This is followed by a delay period of reorganization in which cell aggregates pause in compaction. In the third stage, strong interaction of cadherins is a major factor for the morphological transition from loose cellular aggregates to compact spheroids [44] (Figure 1).

While MSC aggregate cultivation has mainly been conducted in the context of chondrogenic differentiation, it is increasingly used to study cellular behavior under 3D conditions to more closely resemble a physiological setting [45]. Cellular behavior in tissues is determined by diffusive mass transfer, causing gradients of oxygen, nutrients, metabolic waste products, and paracrine mediators [46], which is not appropriately reflected in 2D cultivation. Moreover, MSCs cultivated as aggregates experience a different strain and rigidity during cultivation and adapt their adhesion behavior and phenotype [47] accordingly, potentially resulting in increased immunomodulatory, anti-inflammatory, and angiogenic effects. Due to this increased angiogenic and vasculogenic potential, MSC aggregates might be used as vascularization units and can be considered as building blocks for tissue engineering [48,49].

While there are a number of reviews on the characteristics of MSC aggregates [47,50,51,52,53], the impact of dynamic cultivation on both, MSC aggregates and EVs released from these aggregates remains to be summarized. As physiological pre-conditioning strategies including a 3D environment and dynamic cultivation under hypoxic conditions have been shown to optimize the therapeutic potential of MSCs [50], they may enhance the effects of MSC-derived EVs, but this hypothesis remains to be tested.

## 3. Therapeutic Potential of Aggregate Cultivation

Compared to 2D monolayer cultivation, scaffold-free aggregate cultivation of cells improves their biological properties, resulting in increased cell viability, proliferation, and differentiation, as well as in physiologically relevant metabolism, phenotype, and genotype [20]. For MSC aggregates, in particular, enhanced anti-inflammatory [54], angiogenic, and tissue regenerative effects [55] as well as enhanced differentiation [56], maintenance of stem cell properties and delayed replicative senescence were observed [55,57]. This shift to a more physiological cellular behavior is not only relevant for clinical application, but can also enhance the significance of in vitro models. The following section highlights the therapeutic potential of MSC aggregate cultivation with respect to (1) angiogenic properties, (2) anti-inflammatory properties, (3) immunomodulatory characteristics, (4) stemness, and (5) cell survival and anti-apoptotic properties (Figure 1).

### 3.1. Angiogenic Properties

MSCs cultivated as aggregates exhibit increased angiogenic properties. Human MSC aggregates showed improved therapeutic efficacy for ischemia treatment through increased angiogenic factor secretion which has been attributed to the hypoxic environment inside the aggregates [58]. HGF, VEGF, and FGF-2 levels were 20- to 145-fold higher in medium conditioned with MSC aggregates, as compared to medium from MSCs cultivated in 2D monolayers. Recent studies not only report an improvement in terms of paracrine effects, but also functional improvement through increased neovascularization [59,60], wound healing [60], tube formation, and migration of fibroblasts into a wounded area [61] .

### 3.2. Anti-Inflammatory and Immunomodulatory Effects

Furthermore, MSC aggregates display immunosuppressive effects. Secretion of TNF-α from macrophages decreased in co-culture with MSC aggregates as compared to co-cultivation with a 2D MSC monolayer [54,62]. The secretion of PGE2 [63], HGF [54,63,64,65], and TGF-β [62] which are known to suppress pro-inflammatory markers and direct stimulated macrophages towards an anti-inflammatory phenotype increased upon MSC aggregate cultivation. Likewise, anti-inflammatory factors, such as TNF-α-stimulated gene protein TSG-6 which is known to counteract TNF-α and IL-1 inflammation, were elevated [54]. Furthermore, MSC aggregates suppressed inflammation in a mouse model of zymosan-induced peritonitis [54] and reduced acute kidney injury in a rat ischemia–reperfusion model [66]. The expression of anti-inflammatory and immunomodulatory factors, however, can be further increased by optimizing the microenvironment via the spheroid size, oxygen tension, and inflammatory stimulus [67].

### 3.3. Stemness

During 2D monolayer cultivation, MSCs may undergo aging, loss of clonogenicity, or spontaneous differentiation [68,69]. For clinical application however, it is crucial to maintain the stemness of MSCs during in vitro cultivation. Compared to 2D cultivation, MSC aggregates display increased expression of the pluripotency marker genes *Nanog*, *Sox2*, and *Oct4* [70,71]. miRNAs—namely miR-489, miR-370, and miR-433—which are related to the maintenance of a quiescent adult stem cell state, were highly expressed in MSC aggregates [70], and an increased clonogenicity was observed after aggregate cultivation [70,71]. In a following study, delayed replicative senescence of aggregate-derived MSCs was observed in comparison to monolayer-derived MSCs [55].

### 3.4. Cell Survival and Anti-Apoptotic Effects

The survival of cells after transplantation plays an important role in the therapeutic outcome. As an example, more than 85% of systemically injected MSCs were found in the precapillaries [37]. MSCs cultivated as aggregates displayed better survival in ischemic conditions [72] and higher resistance to oxidative stress-induced apoptosis [73]. Additionally, the pro-apoptotic molecule Bax was downregulated, while the anti-apoptotic molecule Bcl-2 was upregulated in MSC aggregates [57,72], which might contribute to the overall post-transplantation survival of MSCs.

## 4. Generation of MSC Aggregates

**Figure 2:**
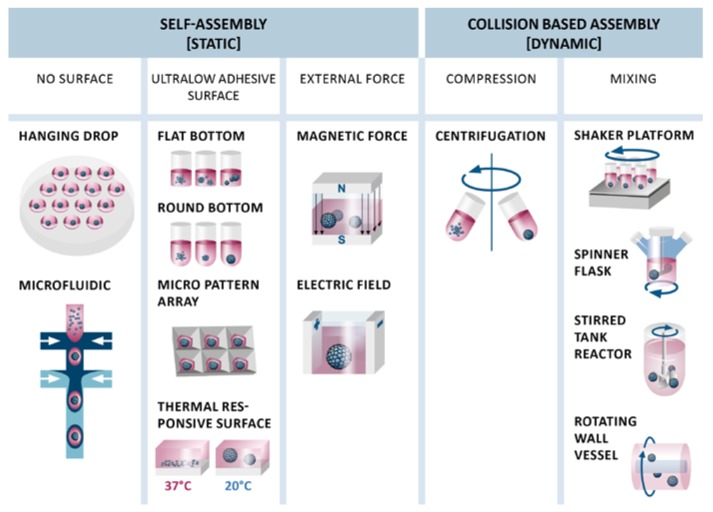
Different techniques for static cluster-based self-assembly and dynamic collision-based assembly of MSC aggregates. Self-assembly of MSCs can be forced using no or ultralow adhesive surfaces or external forces. Collision-based assembly is conducted by compression or mixing.

To generate aggregates, MSC adhesion to tissue culture plates must be avoided. Methods for the generation of aggregates from a single cell suspension can be classified into cluster-based self-assembly and collision-based assembly [74]. Cluster-based self-assembly is a process in a static environment where cells are prevented from attaching to a surface and thus come in contact with each other to form aggregates. In contrast, collision-based assembly takes place in a dynamic environment, where cells collide upon centrifugation or mixing of a single cell suspension (Figure 2).

### 4.1. Static Cluster-Based Self-Assembly

In cluster-based self-assembly, single cells are separated into compartments and undergo the typical three-step process of aggregate formation as shown in Figure 1. Hanging drop cultivation may be the most common cluster-based self-assembly method [49,75,76]. Specialized cell culture plates allow formation of hanging drops from a single cell suspension with subsequent formation of cell aggregates. Beside its labor intensity, the only drawback of this method is that medium changes are challenging and prone to error or destruction of aggregates or the hanging drops. To overcome this limitation, automated [77], robot assisted [78] and microfluidic based [79] high-throughput hanging drop cultivation systems have been developed recently.

Cell culture plates with ultralow adhesive surfaces can be used to generate aggregates, as well [56,62,75]. This method is also referred to as ‘liquid overlay’ method. On flat bottom plates, cells form aggregates of heterogeneous size and shape, whereas aggregate shape and size can be very well controlled in round-shaped cavities, such as round bottom multiwell plates. Based on this principle, different kinds of microwell arrays made from micropatterned agarose [80], polydimethylsiloxane (PDMS) [81] or polyethylene glycol (PEG) hydrogels [82] have been developed to generate large quantities of uniformly sized and shaped aggregates in a cost-effective manner. Other modifications, such as thermally responsive surfaces [83] or polycationic chitosan membranes [71,84], have also been applied to form aggregates. These methods yielded viable aggregates, although heterogeneous in shape and size. Microfluidic systems were also used to generate size controlled aggregates [85]. As an example double-emulsion droplets were used to generate picoliter-sized bioreactors for the self-assembly of MSC spheroids [86]. External forces such as magnetic force [87], electric field [88], or ultrasound wave traps [89] to concentrate cells for aggregation are not as common, and only magnetic force has been used for the aggregation of MSCs so far [90,91].

### 4.2. Dynamic Collision-Based Assembly

Methods for dynamic, collision-based assembly of MSC aggregates include forced aggregation by centrifugation [92] or mixing mediated by shaker platforms [75,93], spinner flasks [56,59], rotating wall vessels (RWVs) [56], and stirred tank reactors (STRs) [94]. Aggregation by centrifugation has mainly been used for chondrogenic differentiation of MSCs [95] and is also known as pellet or micromass culture. Collision-based assembly by mixing was observed with a seeding density of as low as 2 × 10^4^ cells/mL in spinner flasks and RWVs [56], with 1 × 10^5^ cells/mL in a STR [94] and led to randomly sized spheroids, whereas mixing in ultralow adhesive multiwell plates on a shaker platform [75] and compression by centrifugation [92] yielded aggregates with narrower size and homogeneous shape distribution.

## 5. Dynamic Cultivation of Aggregates

**Figure 3:**
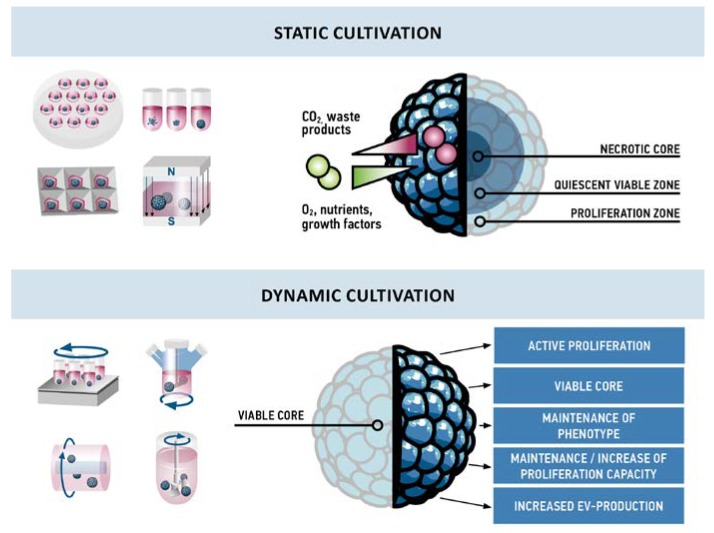
Comparison of effects observed in static and dynamic cultivation of MSC aggregates. Due to gradients of nutrients and waste products, aggregates cultivated under static conditions are usually structured in three layers: a necrotic core in the center, a quiescent viable zone of non-proliferative cells, and an outer layer with proliferating cells. In contrast, dynamic cultivation conditions result in a viable core and active proliferation throughout the aggregate. Cells from these aggregates maintain their phenotype, proliferation capacity, and display an increased production of EVs.

The therapeutic effects of MSCs, MSC conditioned medium, or MSC-derived EVs have been shown to support regeneration after organ and tissue injury. In vitro pre-conditioning strategies can enhance survival, engraftment, and paracrine properties of MSCs and, therefore, optimize their therapeutic potential [50]. Specifically, dynamic cultivation conditions, such as fluid flow have substantial impact on cellular behavior (Figure 3). Increased proliferation, viability, differentiation potential but also paracrine effects were observed in perfusion bioreactors, on horizontal or orbital shaking platforms, or in stirred systems, such as spinner flasks or stirred tank reactors [96,97,98]. However, only a few studies have harnessed dynamic conditions for the generation and cultivation of MSC aggregates (Table 1), as dynamic cultivation aggregates do not necessarily need to be generated by dynamic collision-based assembly. Studies report formation by centrifugation [92], in hanging drops [75], in ultralow adhesive multiwell plates [75], or in a microwell array [82] followed by cultivation in a dynamic cultivation system, such as shaker platforms [75,82,92,93], in spinner flasks [56,59], RWV [56], or STR [94]. In microwell arrays, all seeded cells are involved in the formation of aggregates, thus the size and cell number per aggregate can be precisely controlled.

### 5.1. Proliferation and Viability

Under static conditions, a necrotic core develops with time due to oxygen, nutrition, and waste product gradients along the diameter of an aggregate, and thus aggregates cultivated under static conditions have not been reported to exceed approximately 500 µm in diameter [99,100]. Interestingly, none of the studies on dynamic cultivation of MSC aggregates reported a necrotic core inside the aggregates. In contrast, two studies reported active proliferation at the core of the aggregates [92,93], and an up to six-fold expansion of cells was observed [93,94]. Increased convection in dynamic cultivation seems to improve oxygen and nutrient supply and to inhibit the formation of gradients. In contrast, Cha et al. observed active proliferation only in 3D static conditions whereas in 3D dynamic conditions cells seemed to rest, although they were more active in terms of EV production [82]. The different cellular behavior might not only be owed to different aggregate formation and cultivation techniques but also to different media compositions. High expansion was observed in optimized serum-free [93] or platelet lysate supplemented [94] medium, whereas less or no proliferation was observed using fetal bovine serum-containing medium [82].

### 5.2. Stemness

Mechanical stimulation by shear forces during dynamic cultivation can trigger spontaneous differentiation of MSCs and thus compromise their stemness. Therefore, surface marker expression and differentiation capacity of MSCs have been evaluated in previous studies after dynamic aggregate cultivation. None of the studies analyzing surface marker expression according to the guidelines of the ISCT found alterations of the phenotype [56,59,93,94]. However, during aggregate formation and cellular reorganization mesenchymal stem cell markers seemed to be altered [56,82]. When cells were cultivated for an extended period as aggregates or were dissociated after 3D dynamic cultivation and cultivated again in 2D models, cells expressed a typical MSC phenotype. Also, MSC aggregates were kept in an undifferentiated state over a period of 16 days on a shaker platform at 45 rpm [92] and for a period of 6 days at 600 rpm in a STR with an average shear stress of 0.2 Pa [94]. Thus, aggregate formation might shield the inner cell mass from shear forces and helps to avoid spontaneous differentiation. Regarding differentiation capacity all studies that either differentiated MSC aggregates [56,75,92] or differentiated MSCs after 3D dynamic cultivation [93,94] observed robust trilineage differentiation as analyzed by histological stainings and/or gene expression. The studies testing differentiation of MSC aggregates against 2D static observed increased adipogenic and osteogenic differentiation [56,75,93]. However, it remains unclear if the cultivation under 3D dynamic conditions increases the differentiation potential in comparison to 3D static cultivation.

### 5.3. Therapeutic Potential

In the context of therapeutic potential, a direct comparison between dynamic and static cultivation of 3D aggregates would be of interest since until now the gene regulation of 3D dynamic cultivated cells was only compared to 2D static cultivation. The study observed 710 genes that were differently expressed (277 downregulated and 433 upregulated genes). The most differently expressed genes were classified under (1) biological adhesion, structural molecule activity, and ECM which pointed to changes in the cytoskeleton; (2) developmental process which affected numerous secreted factors like IL-24; and (3) hypoxia related genes [56]. Also, the secretion of angiogenic factors like VEGF, HGF, and HGF-2 was significantly increased in an ischemic limb model after dynamic cultivation when cells were grafted as spheroids compared to dissociated cells [59]. The same study observed a higher survival rate after transplantation. Furthermore, the production of EVs was strongly increased during dynamic aggregate cultivation compared to static aggregates [82] and cytokine levels in these EVs was significantly higher. These findings suggest that dynamic cultivation of MSC aggregates might increase the therapeutic potential of MSCs or of MSC-derived EVs.

## 6. Concluding Remarks

Only few studies so far have addressed dynamic cultivation of MSC aggregates and since different techniques for the generation and cultivation were used, results are diverse and not directly comparable. Moreover, until now only Cha et al. specifically compared 3D static to 3D dynamic cultivation of MSC aggregates [82]. However, existing reports on the dynamic cultivation of MSC aggregates highlight the maintenance of stemness, improved differentiation capacity, and to some extent active proliferation of cells (Figure 3).

The dynamic cultivation of MSC aggregates might be a suitable strategy to develop a passage-free expansion system. Since convection reduces oxygen, nutrient and waste gradients along the diameter of aggregates, larger viable aggregates without necrotic cells in their core can be generated under dynamic conditions. Currently, the growth rate is not competitive to microcarrier-based expansion systems [101,102]. Up to now, however, no study has investigated the cellular growth in dynamically cultivated MSC aggregates for more than seven days, thus after optimization MSC aggregates might be a viable option for in vitro expansion. Interestingly, up to six-fold expansion of MSCs was observed within seven days with a medium optimized for dynamic aggregate cultivation of MSCs [93] indicating further optimization potential.

Also, defined starting conditions in terms of uniform aggregate shape, size, and cell number are needed in future studies. As more manufacturers offer ready-to-use micropatterned multiwell plates that do not need additional centrifugation steps or rinsing agents, this might be the best option to generate lager numbers of uniform spheroids at low cost and a minimum of time.

Next to relevant biological implications and advantages of the 3D culture, the large number of clinical studies (>700 from www.clinicaltrials.gov) based on MSC demand for novel conditions where cells may be cultured in a more cost-effective manner. This could allow the reduction of MSC manufacturing costs, contributing to the progress towards larger phase III studies and, ultimately, to a wider diffusion of their therapeutic potential. 

Although more studies on the direct comparison of 3D static to 3D dynamic cultivation of MSC aggregates are needed, the upregulation of hypoxic genes which results in increased angiogenesis, the upregulation of the cancer suppressing cytokine IL-24, and the increased EV production found in dynamically cultivated MSC aggregates may be promising for future clinical application. Due to this high therapeutic potential, the large-scale production of MSC-EVs and their standardization is becoming a crucial issue for clinical translation [103,104,105].

## Figures and Tables

**Table 1:** Comparison of different studies reporting on dynamic cultivation conditions for the cultivation of MSC aggregates.

Ref.	Cultivation System	Cells Per Spheroid	Initial Cell Density (c/mL)	Rotation (rpm)	Duration (days)	Surface Marker	Differentiation Capacity	Effects	Aggregate Size (µm)
[56]	SF and cultivation in spinner flask	random	2 × 10^4^	30	7	~	A↑, O↑ (compared to 2D static)	Hypoxia-linked genes ↑, changes in ECM organization, IL24↑	56–135 (avrg. 99)
[56]	SF and cultivation in rotating wall vessel bioreactor	random	2 × 10^4^	15	7	~	A↑, O↑ (compared to 2D static)		18–44 (avrg. 32)
[75]	SF and cultivation on orbital shaker	random	5 × 10^4^	95	Aborted after 3 days	-		-	Multi aggregation
[75]	Formation in hanging drop, cultivation in suspension on orbital shaker	5000	2.5 × 10^5^	95	Aborted after 3 days	-		-	Multi aggregation
[75]	96-well plate on orbital shaker followed by static cultivation	1–2 × 10^4^	0.6–1.3 × 10^5^	95	2 dynamic followed by 21 static	-	O↑ (compared to control)	Col1, Col3, OPN, BMP-2↑	200
[59]	SF and cultivation in spinner flask	random	6 × 10^5^	70	3	~	-	Anti-apoptotic, angiogenic factors, preservation of ECM, enhanced survival after transplantation	100–350
[92]	Formation by centrifugation followed by orbital shaker	300/600/1000	1.8–6 × 10^6^	45	21	-	A↑, O↑ (compared to 2D static)	Active proliferation in the center of the spheroid, undifferentiated up to 16 days,	157/100/177 (day 7)
[93]	SF and cultivation in shaker flask on horizontal shaker	random	1 × 10^5^	80	7	~	A, C, O (dissociated cells in 2D static after 3D dynamic)	Active proliferation in the center of the spheroid, up to 6-fold expansion	-
[94]	SF and cultivation in stirred tank bioreactor	random	1 × 10^5^	600	6	~	A, C, O (dissociated cells in 2D static after 3D dynamic)	Approx. 2-fold expansion	-
[82]	Microwell array on orbital shaker	400	5 × 10^5^ cells/array	30	7	-	-	No proliferation, EV production ↑	150

SF: spontaneous formation of aggregates, A: adipogenic differentiation, C: chondrogenic differentiation, O: osteogenic differentiation, decrease: ↓, increase: ↑, comparable to 2D: ~, not measured: -.

## Abbreviations

2D	Two-dimensional
3D	Three-dimensional
ECM	Extracellular matrix
EGF	Epidermal growth factor
EVs	Extracellular vesicles
FGF	Fibroblast growth factor
GvHD	Graft-versus-host disease
HRMPs	High risk medicinal products
IGF	Insulin-like growth factor
ISCT	International Society for Cellular Therapies
MSCs	Mesenchymal stem cells
PDGF	Platelet-derived growth factor
PDMS	Polydimethylsiloxane
PEG	Polyethylene glycol
PGE2	Prostaglandin E2
RWV	Rotating wall vessel
SCDF-1	Stromal cell-derived factor 1
STR	Stirred tank reactor
TGF-β	Transforming growth factor-β
VEGF	Vascular endothelial growth factor
